# Genome-wide perturbations by miRNAs map onto functional cellular pathways, identifying regulators of chromatin modifiers

**DOI:** 10.1038/npjsba.2015.1

**Published:** 2015-09-28

**Authors:** Tyler J Moss, Zijun Luo, Elena G Seviour, Vasudha Sehgal, Yiling Lu, Steven M Hill, Rajesha Rupaimoole, Ju-Seog Lee, Cristian Rodriguez-Aguayo, Gabriel Lopez-Berestein, Anil K Sood, Robert Azencott, Joe W Gray, Sach Mukherjee, Gordon B Mills, Prahlad T Ram

**Affiliations:** 1 Department of Systems Biology, The University of Texas MD Anderson Cancer Center, Houston, TX, USA; 2 Medical Research Council Biostatistics Unit, Cambridge, UK; 3 Department of Gynecologic Oncology, The University of Texas MD Anderson Cancer Center, Houston, TX, USA; 4 Department of Experimental Therapeutics, The University of Texas MD Anderson Cancer Center, Houston, TX, USA; 5 Center for RNAi and Non-Coding RNA, The University of Texas MD Anderson Cancer Center, Houston, TX, USA; 6 Department of Mathematics, University of Houston, Houston, TX, USA; 7 Department of Biomedical Engineering, Oregon Health & Science University, Portland, OR, USA; 8 Cancer Research UK Cambridge Institute, School of Clinical Medicine, University of Cambridge, Robinson Way, Cambridge, UK

## Abstract

**Background:**

Regulation of gene expression by microRNAs (miRNAs) is critical for determining cellular fate and function. Dysregulation of miRNA expression contributes to the development and progression of multiple diseases. miRNA can target multiple mRNAs, making deconvolution of the effects of miRNA challenging and the complexity of regulation of cellular pathways by miRNAs at the functional protein level remains to be elucidated. Therefore, we sought to determine the effects of expression of miRNAs in breast and ovarian cancer cells on cellular pathways by measuring systems-wide miRNA perturbations to protein and phosphoproteins.

**Methods:**

We measure protein level changes by reverse-phase protein array (RPPA) in MDA-MB-231, SKOV3.ip1 and HEYA8 cancer cell lines transfected by a library of 879 human miRNA mimics.

**Results:**

The effects of multiple miRNAs–protein networks converged in five broad functional clusters of miRNA, suggesting a broad overlap of miRNA action on cellular pathways. Detailed analysis of miRNA clusters revealed novel miRNA/cell cycle protein networks, which we functionally validated. *De novo* phosphoprotein network estimation using Gaussian graphical modeling, using no priors, revealed known and novel protein interplay, which we also observed in patient ovarian tumor proteomic data. We identified several miRNAs that have pluripotent activities across multiple cellular pathways. In particular we studied miR-365a whose expression is associated with poor survival across several cancer types and demonstrated that anti-miR-365 significantly reduced tumor formation in animal models.

**Conclusions:**

Mapping of miRNA-induced protein and phosphoprotein changes onto pathways revealed new miRNA-cellular pathway connectivity, paving the way for targeting of dysregulated pathways with potential miRNA-based therapeutics.

## Introduction

Noncoding RNAs, such as microRNAs (miRNAs), mediate epigenetic control of gene expression, generating diverse, complex phenotypes. miRNAs are small (~22 nt) RNAs that bind to and regulate translation or degradation of cognate mRNA. The binding of a given miRNA to its target mRNA occurs via the protein machinery in the RNA-induced silencing complex and alignment of a seven- to eight-nucleotide sequence (seed sequence) to the complementary sequence in the target mRNA. Owing to the brevity of the seed sequence and regularity of imperfect alignment, a single miRNA can regulate many (sometimes >100) mRNAs. Given their promiscuity, miRNAs are important regulators of gene expression in multiple cellular pathways, and dysregulation of miRNA expression can have severe consequences on cell behavior, leading to the development of diseases, such as cancer. Several miRNAs have been shown to have important roles in tumorigenesis either when overexpressed (e.g., miR-21)^1^ or via loss of inhibition of tumor progression when downregulated (e.g., miR-34a, let-7).^2,3^

Major research efforts over the past decade have focused on the function of miRNAs and their target mRNAs. The investigators in these studies predominantly relied on changes in mRNA and target prediction to determine the role of given miRNAs in cells. However, miRNA-mediated silencing of gene expression via inhibition of protein translation can occur without a measureable decrease in mRNA expression. Furthermore, mRNA measurements are insensitive to the indirect impact of miRNAs on protein stability and/or activity. Indeed, overall mRNA expression levels may be weak predictors of protein levels and activity.^4,5^ Therefore, considering changes in protein levels is important when validating miRNA targets particularly when characterizing the downstream functional consequences of miRNAs. In the present study, we determined the effects of genome-wide perturbations by miRNAs on protein levels in cancer pathways by introducing 879 miRNA mimics into cancer cell lines and measuring changes in 127 proteins and phosphoproteins in these cells. We characterized miRNAs according to their roles in regulating protein and phosphoprotein networks and cellular pathways. Using advanced clustering and machine-learning techniques, we isolated important clusters of miRNAs and corresponding sets of regulated proteins. We used probabilistic graphical modeling, using no prior connectivity information, to construct *de novo* networks that recapitulated well characterized protein–protein interactions as well as identifying interplay between proteins across different pathways. We identified important regulatory miRNAs by classifying the miRNAs based on their effects on cellular functions as well as identifying more broadly acting miRNAs that appear to alter multiple pathways. Further analysis of one such miRNA, mir-365a, identified its role in regulating epigenetic modification of chromatin and its dysregulation in multiple cancers. We demonstrated that targeting miR-365a with anti-miR-365 resulted in significant reduction of tumors in a xenograft model.

## Materials and Methods

### miRNA screens

A miRNA library screen was performed using the reverse phase protein array (RPPA) platform.^6,7^ Screening by RPPA enabled us to directly measure changes in protein levels as well as post-translational protein modifications. By RPPA, we screened 1,000 samples per chip probing for proteins limited to the number of available high-quality antibodies. The primary screen was performed in MDA-MB-231 cells using 879 miRNAs measuring changes in 127 proteins (see Supplementary Table 2 for antibody list). A subset of the miRNA library (154 miRNAs) was used in a second screen using three cell lines (see Supplementary Tables 2 and 3 for lists of antibodies and miRNAs, respectively) measuring 127 proteins, 103 of which were common to the first screen. A cell proliferation screen of miRNA library was also performed in MDA-MB-231 cells using the Cell Titer Blue Cell Viability Assay Reagent (Promega, Madison, WI, USA). See Supplementary Methods for screen and data normalization details.

### Microarray

Gene expression measurements of miR-365a-treated SKOV3.ip1 and MDA-MB-231 cells were performed using HumanHT-12 v4 Expression BeadChips (Illumina, San Diego, CA, USA). Data were quantile normalized using the lumi Bioconductor package^8^ and are available in the Gene Expression Omnibus: GSE64020.

### Mouse xenograft tumor model

Twenty mice were given intraperitoneal injections of SKOV3 cells (10^6^ cells/mouse). After 7 days the mice were treated with intraperitoneal injections of miRNA encapsulated in liposomes (5 μg/mouse in 200 μl of PBS) twice a week until mice became moribund in either group. Half of the mice were treated with a control miRNA hairpin inhibitor and the other half were treated with the miR-365a hairpin inhibitor. The mice were killed 30 days after the initial injection and the mouse body weight, tumor weight, tumor nodule count, location of metastases and presence of ascites was measured. All animals were cared for according to guidelines set forth by the American Association for Accreditation of Laboratory Animal Care and the US Public Health Service policy on Human Care and Use of Laboratory Animals. See Supplementary Information for more details.

For other detailed methods see the Supplementary Methods.

## Results

### Functional classification of miRNAs

**Figure 1:**
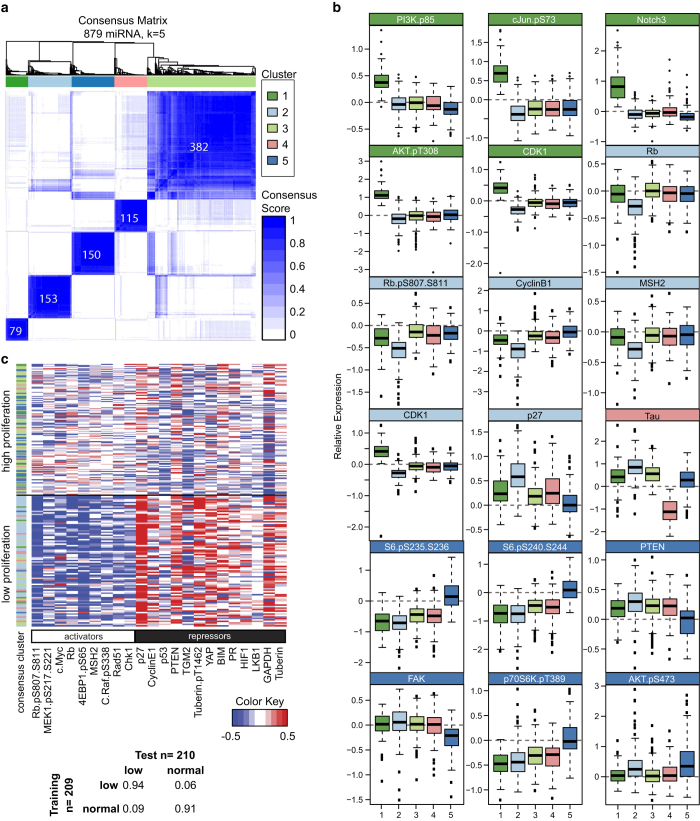
Consensus clustering of miRNAs according to their functional roles in regulating cancer pathways. (**a**) Heatmap of miRNA consensus clusters and the number of miRNAs in each cluster. (**b**) Box-whisker plots of discriminatory proteins in the first screen regulated by miRNA clusters. Cluster 1 miRNAs (dark green) upregulated PI3K/AKT, Notch and c-Jun signals. Cluster 2 miRNAs (light blue) regulated the levels cell cycle proteins. Cluster 4 miRNAs (pink) downregulated Tau levels. Cluster 5 miRNAs (dark blue) upregulated the mTOR pathway. (**c**) Heatmap of the fold change in levels of protein activators and repressors. Each row represents a miRNA-transfected observation, and each column represents a protein. The miRNAs are grouped according to their effect on cell proliferation. Table inset, confusion matrix of a SVM model predictors of cell number changes.

To determine the impact of miRNAs on cancer pathways, we perturbed the basal-like breast cancer cell line MDA-MB-231 (KRAS G13D, BRAF G464V, TP53 R280K and CDKN2A frameshift mutations) with 879 exogenous miRNA mimics and changes in protein levels were measured by RPPA. Well-characterized miRNAs, such as members of the let-7, miR-200 and miR-17-92 families, cocluster using unsupervised hierarchical clustering (Supplementary Figure 2) showing that miRNAs from the same families have a similar, protein-level effect in our screen. We clustered the miRNAs into functional groups by their similarity in inducing changes in levels of protein found in cancer pathways (Supplementary Figure 1) using the ConsensusClusterPlus package in the statistical computing environment R.^9^ The miRNAs were clustered by k-means with numbers of clusters ranging from 2 to 15, repeated 100 times, leaving out 10% of the miRNAs in each repetition. We grouped the miRNAs by the frequency with which they clustered together. We chose five as the optimal number of functional clusters (Supplementary Figure 3) and each miRNA was classified into one of them (Figure 1a). We then identified proteins defining each of the clusters, based on the ability of the proteins to differentiate the miRNAs in each consensus cluster from the miRNAs in all other consensus clusters using random forest classification. The most important discriminatory proteins for each miRNA cluster are shown in the box-whisker plots in Figure 1b.

The 79 miRNAs in functional cluster 1 (Figure 1a,b) are characterized by shared upregulation of Notch3 and activation of the phosphoinositide 3-kinase (PI3K)/AKT pathway as well as activation of c-Jun. Functional cluster 2 contains 153 miRNAs that are enriched in targeting regulators of the cell cycle and proliferation. The 115 miRNAs in cluster 4 are strongly associated with downregulation of Tau protein levels. The 150 miRNAs in functional cluster 5 are associated with decreased PTEN levels and concurrent upregulation of the Akt/p70S6K/S6 pathway. The remaining 382 miRNAs have relatively minor effects on the proteins assessed and are grouped in cluster 3 (Figure 1b).

Large screens inherently produce false-positive and -negative results. To validate the findings from our primary screen, we performed a secondary screen using a subset of the miRNAs from all of the clusters (Supplementary Table 1). We performed the secondary screen with MDA-MB-231 cells as well as the ovarian cancer cell lines HeyA8 and SKOV3.ip1. We rescreened 154 miRNAs in each of the three cell lines. We tested the consistency of regulation of protein levels in the first screen and in the MDA-MB-231 cells in the second screen using Markov transition matrix analysis, wherein the fraction of upregulated and downregulated proteins for each miRNA in both screens was determined (Supplementary Figure 4a). Analysis of the sum of the transitions across all proteins for all of the common miRNAs in both screens demonstrated a 78% overlap of true-positive changes in the MDA-MB-231 cell line in the two screens (Supplementary Figure 4b). We observed that several proteins and miRNAs exhibited robust changes in the two MDA-MB-231 screens as well as across all three cell lines (Supplementary Figure 5). The changes in cell cycle proteins were the most consistent in the two screens; hence, the miRNAs in functional cluster 2 exhibited the most consistency in the two screens and across the three cell lines (Supplementary Figure 4c). However, cluster 4 miRNAs that were characterized by changes in Tau protein levels, demonstrated poor correlation in the MDA-MB-231 cells in the first and second screens, suggesting that this subset of cluster 4 miRNAs selected for the second screen may not represent those that are *bona fide* regulators of Tau expression.

### Support vector machine and predictors of cellular proliferation

In addition to the changes in protein and phosphoprotein levels in response to perturbation by miRNAs, we measured changes in MDA-MB-231 cell number 48 h after transfection with the miRNAs. Using average correlation analysis (see Supplementary Methods) between protein levels and changes in cell number, we identified activators and repressors of proliferation (Supplementary Figure 6). We examined miRNA induced changes in cell number relative to nontargeting miRNA controls and binned the miRNA into high proliferation (cell-number change >5% of control values) and low proliferation (lowest 10%) groups. The data demonstrated that 14% of the miRNAs did not change the cell numbers. We analyzed the data using a machine-learning algorithm (support vector machine) to predict changes in cell number as a function of changes in protein and phosphoprotein levels using the 210 miRNAs in the low-proliferation (*n*=88) and high-proliferation (*n*=122) groups. Assessment of the support-vector machine confusion matrix analysis, predicting changes in cell number using leave-one-out cross-validation demonstrated >90% accuracy (Figure 1c). When we grouped the miRNA-perturbed samples according to the highest and lowest quantiles of proliferation, it revealed an apparent pattern in which the levels of the predicted activators of proliferation were downregulated and the proliferation repressors were upregulated (Figure 1c). Cluster 2 miRNAs make up a preponderance of the miRNAs belonging to the low proliferation group and exhibit a pattern of protein levels consistent with downregulating proliferation.

### Cluster 2 miRNAs regulate proliferation by altering cell cycle proteins

**Figure 2:**
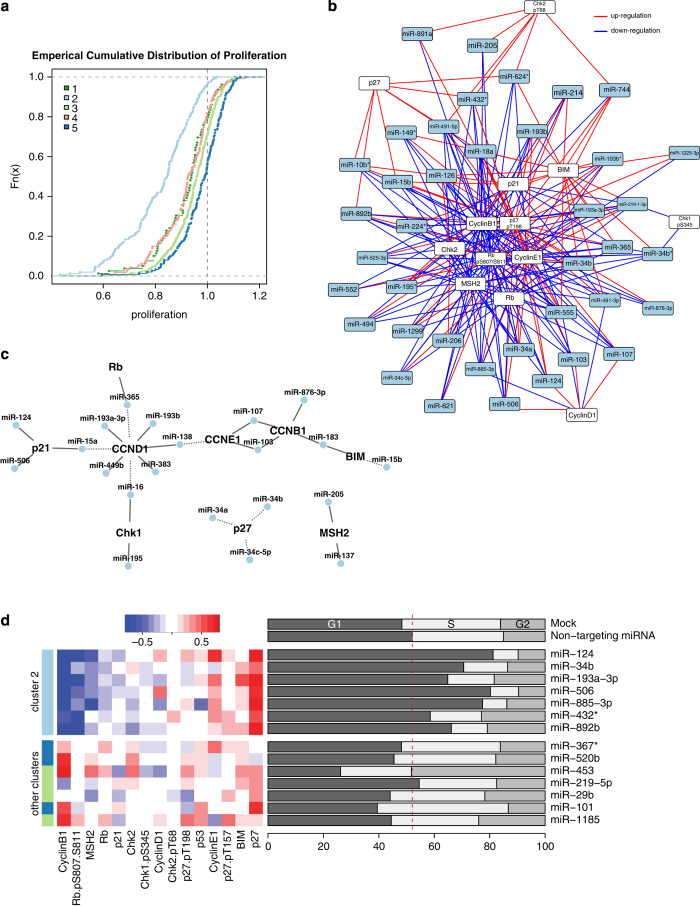
Regulation of cell cycle proteins and induction of decreased proliferation of cancer cells by cluster 2 miRNAs. (**a**) Distribution of the relative change in cell number for all miRNAs grouped by cluster. Cluster 2 miRNAs decreased the number of MDA-MB-231 cells after 48 h more so than did miRNAs in the other clusters. (**b**) Network of miRNA regulators of cell cycle proteins. (**c**) Direct interactions network of miRNA of cell cycle proteins. Each miRNA–protein edge is a predicted interaction with observed changes in our screen. Solid edges indicate down-regulation of protein levels and solid edges indicate upregulation. (**d**) Heat map and cell cycle distributions of representative miRNAs in cluster 2.

Several pathways regulate cell cycle proteins, which result in alterations in cellular proliferation. The distribution of proliferation values grouped by functional miRNA clusters demonstrated that consensus cluster 2 miRNAs markedly downregulated cellular proliferation over that induced by control miRNAs and the miRNAs in all other functional clusters (Figure 2a). Individually and as a whole, the cluster 2 miRNAs downregulated the levels of positive regulators and upregulated the levels negative regulators of cell cycle progression (Figure 2b). Several of the miRNAs are predicted to target the cell cycle proteins directly (Figure 2c). Many of the miRNAs that regulate the cell cycle protein levels and that are predicted to target the mRNA of these proteins share identical seed sequence (e.g. miR-124 and miR-506 have identical seed sequence and both downregulate p21). To determine whether cluster 2 miRNAs can induce cell cycle arrest, we selected 14 miRNAs—seven in cluster 2 and seven in other clusters—and measured cell cycle distributions of MDA-MB-231 cells transfected with these miRNAs. The cluster 2 miRNAs induced an increase in the number of cell in G1-phase over that induced by the other miRNAs (Figure 2d). Cluster 2 miRNAs resulting in functional cell cycle arrest at G1 phase was consistent with the regulation of cell cycle proteins by this cluster of miRNAs that we observed in our proteomic screening.

### *De novo* phosphoprotein networks recapitulate known signaling pathway interactions

**Figure 3:**
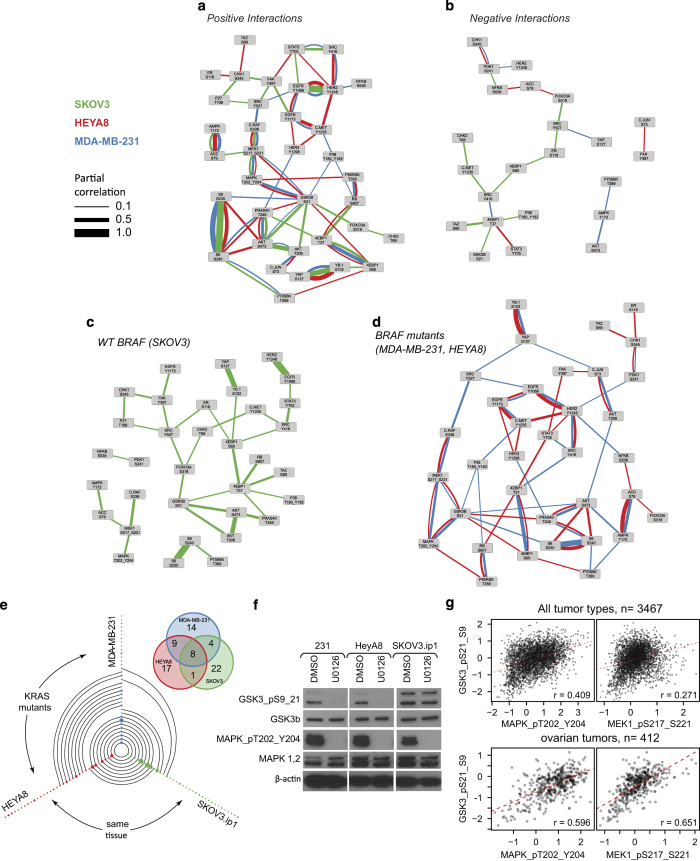
*De novo* phosphoprotein networks. (**a**,**b**) Positive and negative interplay between phosphoprotein components of signaling networks as estimated by graphical network modeling of miRNA perturbation data specific to the cell lines shown. (**c**,**d**) Differences in signaling networks between cell lines with differing KRAS and BRAF mutational statuses. (**e**) Hive plot of the phosphoprotein links in each cell line (node) and the common links among the cell lines (edges). Inset, Venn diagram of the common links across all three cell lines. (**f**) Western blot analysis of the effect of the MEK inhibitor U0126 on ERK and GSK3β phosphorylation in the three cell lines. (**g**) Scatter plots demonstrating correlation between MEK, ERK, and GSK3β phosphorylation in The Cancer Genome Atlas (TCGA) pancancer and ovarian tumor samples.

miRNA can modulate signaling networks by directly or indirectly regulating the abundance of protein components of networks or by regulating the activators or repressors of signaling activity. Furthermore, because proteins and phosphoproteins are components of signaling pathways, such miRNA-mediated changes in protein levels and protein activity may be propagated via the signaling networks, resulting in downstream positive or negative changes in other proteins in the networks. This led us to hypothesize that miRNA perturbation of phosphoprotein networks may provide valuable information for *de novo* identification of signaling networks. To test this hypothesis, we used computational network inference to identify signaling networks using the data from the three cell lines in our second screen. The relatively large number of miRNAs (154 per cell line) allowed for inference of networks specific to each cell line. We modeled cell line-specific phosphoprotein networks using a Gaussian graphical model,^10^ estimated using the ‘graphical lasso’ algorithm (Figure 3a,b; edge widths represent edge scores or partial correlations).^11^ This approach quantifies interplay between pairs of phosphoproteins while taking into account the effects of other measured phosphoproteins (partial correlations). Without the use of any prior information or explicit modeling of the perturbations, this *de novo* network analysis revealed network connectivity that was in remarkable agreement with consensus phosphoprotein pathways and networks as described in the biochemical literature (http://stke.sciencemag.org/cm/), capturing known signaling interactions such as those between EGFR and ErbB2, Raf-MEK-MAPK, AKT and PRAS40, AMPK and ACC, and c-Src and STAT3. This analysis also identified associations that have not been well characterized,^12,13^ such as those between MAPK1/2, MEK and GSK3β.

To validate whether such a functional interaction, or link, exists between MEK, MAPK1/2 and GSK3β, we experimentally manipulated MEK activity and measured GSK3β phosphorylation and observed that MEK inhibition decreased GSK3β phosphorylation in the cells (Figure 3f). We observed the functional associations between MEK, MAPK1/2, and GSK3β in the two cell lines with low to no basal AKT activity (HeyA8 and MDA-MB-231) but not in SKOV3.ip1, which has PTEN loss and PIK3CA mutation and thus high AKT phosphorylation levels (Figure 3c,d) where the interplay may be masked due to the high basal activity of AKT on GSK3b. We also examined the significance of the functional relationship between MAPK1/2 and GSK3β phosphorylation in primary human tumor samples. Pearson correlation analysis of data obtained from The Cancer Genome Atlas revealed a significant positive correlation between phosphorylated MAPK1/2 and phosphorylated GSK3β (*r*=0.409 across all tumors and *r*=0.596 for ovarian tumors) and between phosphorylated MEK and phosphorylated GSK3β (*r*=0.271 across all tumors and *r*=0.651 for ovarian tumors), suggesting that interplay between MEK signaling and GSK3β phosphorylation can be manifested in clinical tumor samples (Figure 3g).

Whereas some of the stronger functional links are common to all cell lines, many phosphoprotein associations are unique to only one or two cell lines. Thus, many *de novo* functional phosphoprotein associations may be mutation specific and context specific. Analysis of our perturbation data identified eight functional links common to MDA-MB-231, HeyA8 and SKOV3.ip1 cell lines and these links have some of the highest partial correlation values (Figure 3e). The two KRAS/BRAF mutant cell lines (MDA-MB-231 and HeyA8) had nine common links that were not present in the cell line wild-type for KRAS and BRAF (SKOV3.ip1). In addition, the two ovarian cancer lines had only one shared link, whereas the breast cancer cell line did not have this link (Figure 3e). This suggests that the interplay in signaling networks may be more sensitive to the underlying mutational background than to the tissue of origin, this requires further investigation.

### miRNA regulators of phosphoprotein networks

**Figure 4:**
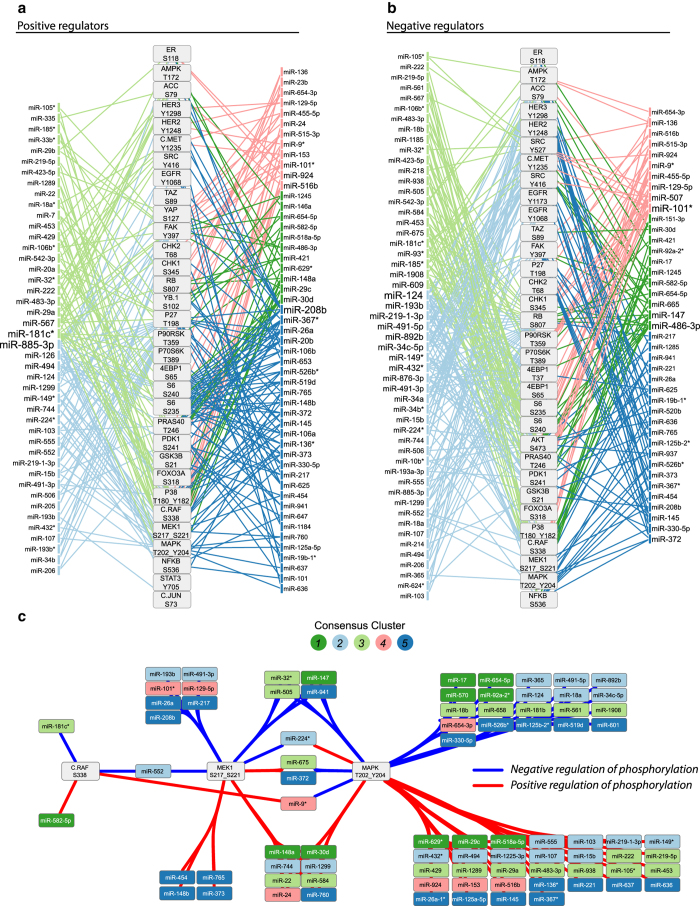
MiRNA regulators of phosphoprotein networks. Negative (**a**) and positive (**b**) miRNA regulators of phosphoprotein are shown. The miRNA-phospho protein edges were determined according to the secondary screen wherein an miRNA markedly downregulates or upregulates a phosphoprotein in at least two of the three cell lines. Only those miRNAs that regulated more than one phosphoprotein in the network are displayed in **a** and **b**. Edges are colored by miRNA cluster and the miRNA label font is sized relative to the number of phosphoproteins it regulates. (**c**) Network of positive and negative miRNA regulators of phosphorylation in Raf-MEK-ERK signaling. Edges are determined as in **a** and **b**. The nodes are grouped and the edges bundled by common regulation of the ERK signaling module.

As described above, we observed that perturbations of phosphoprotein networks by miRNAs can reveal functional connectivity of the underlying networks. We then investigated the miRNAs that functionally regulate proteins in the networks to identify the miRNAs that are the most efficient in perturbing signaling processes. We first identified miRNAs that upregulated the phosphorylation of proteins in the networks using the fold change from the second screen. Figure 4a shows a network of the phosphoproteins measured in the three cell lines and the miRNAs that functionally regulated at least two phosphoproteins in the network (edges are colored by miRNA cluster). Cluster 2 miRNAs (light blue) overwhelmingly upregulated p27 phosphorylation and cluster 5 miRNAs upregulated S6 phosphorylation consistent with that which was observed for other members of their respective clusters in screen 1. MiR-885-3p, miR208b and miR-181c* regulated the highest number of components within the phosphoprotein network.

Figure 4b shows the integrated phosphoprotein network and the miRNAs that negatively regulated individual phosphoproteins in the network. Cluster 2 miRNA downregulate retinoblastoma 1 (RB) phosphorylation and surprisingly regulate many RTKs in the phospho network. MiR-124, miR-101*, miR-193b and miR-486-3p were the most important miRNAs in negatively regulating the network in terms of connectivity. For signaling modules, such as Raf/MEK/ERK, where there was not a clear pattern of regulation by any single cluster of miRNAs we were able to identify miRNAs that regulate single or multiple components of the subnetwork (Figure 4c). The identification of this functional miR-phoshoprotein interaction network is important for the characterization of miRNA-regulated protein networks.

### Functional regulation of cellular pathways

**Figure 5:**
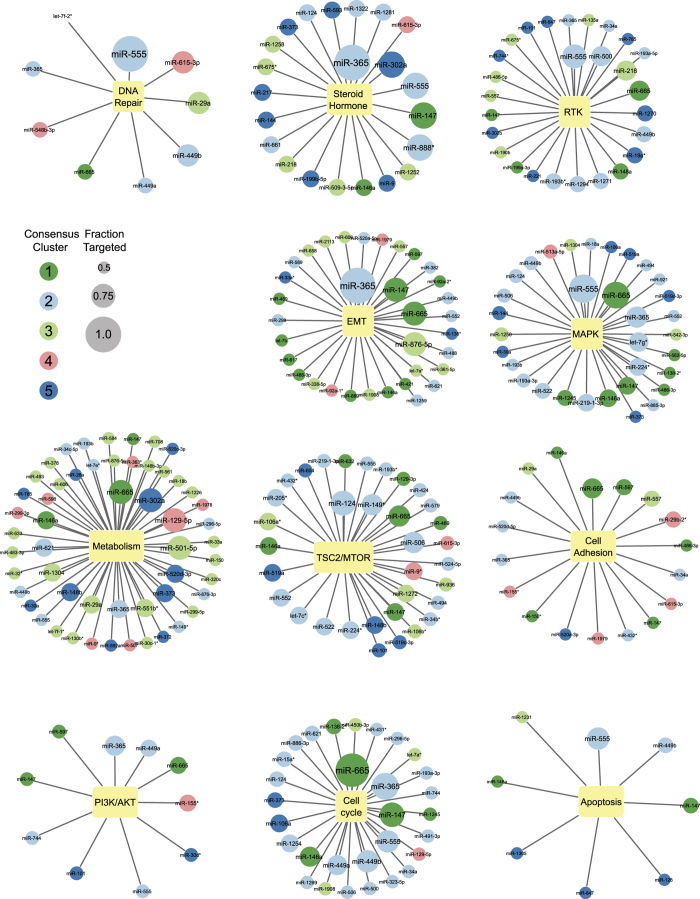
Key miRNA regulators of cancer pathways. Networks of miRNAs that regulate >50% of the proteins in given functional pathways are shown. The miRNAs are colored by consensus cluster, and the miRNA nodes were scaled according to the fraction of proteins regulated in the pathway.

We sought to identify the top miRNA regulators of each of the cancer pathways represented by the proteins measured by RPPA. We separated the RPPA proteins and phosphoproteins into functional pathways (Supplementary Figure 1) as described previously,^4^ allowing for proteins to be included in more than one pathway. We identified the miRNAs regulating each protein using the discretized data from the first screen. Also, we determined the fraction of regulated pathway components for each miRNA and generated networks for all of the miRNAs regulating at least half of the nodes in each pathway (Figure 5). We weighted both node size and edge length according to the fraction of the pathway proteins regulated by each miRNA.

Analysis of the pathways regulated by miRNAs in the functional clusters demonstrated that 63% of the miRNAs regulating the cell cycle proteins belonged to cluster 2. Cluster 2 miRNAs also made up the largest percentage of miRNAs regulating the MAPK (48%), TSC2/mTOR (50%), DNA repair (44%) and PI3K/AKT (40%) pathways. Also, proteins in the metabolism pathway were regulated by 54 miRNAs, 24 (44%) of which belonged to cluster 3.

The RPPA antibody set included antibodies that detect cleaved caspases and protein regulators of apoptosis which can be used to estimate activation of apoptosis. We identified potential miRNA regulators of apoptosis including miR-449b which has been reported to induce apoptosis in gastric cancer.^14^

We also determined the individual miRNAs that have the greatest functional impact on across many pathways. We found that miR-365 functionally regulated the most proteins in steroid hormone, EMT, cell cycle, PI3K/AKT and MAPK pathways. Furthermore, miR-124 coordinately regulated TSC2/mTOR and cell cycle proteins. MiR-665 was one of the top regulatory miRNAs in multiple pathways in our screen, regulating the levels of proteins in the metabolism, MAPK, RTK, TSC2/mTOR, cell adhesion, PI3K/AKT, cell cycle and DNA repair pathways. In addition, miR-555 was highly linked with the DNA repair, steroid hormone, RTK, MAPK, and apoptosis pathways. Despite having marked effects on the majority of proteins of cellular pathways in our functional screen, miR-555 and miR-665 are relatively understudied, with limited reports describing them in the literature.

### MiR-365a regulates chromatin modifiers

**Figure 6:**
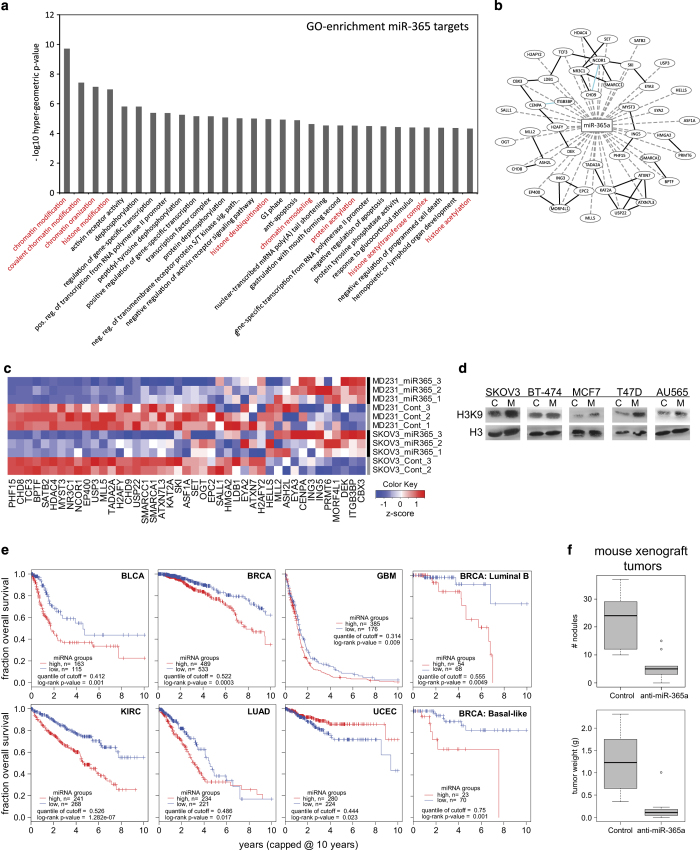
MiR-365a-3p regulates chromatin modifiers and is associated with poor outcome of cancer patients. (**a**) Gene ontology-based analysis of mRNA targets of miR-365a-3p. These are predicted mRNA targets with evidence of binding by the argonaute from AGO-CLIP-seq data from multiple cell lines. (**b**) Network of predicted and validated targets of miR-365a-3p. (**c**) Heat map of the fold change in expression of genes in **b** after transfection of SKOV3.ip1 and MDA-MB-231 cell lines with miR-365a-3p. (**d**) Increased histone H3 acetylation of lysine 9 in cell lines after miR-365a-3p transfection. (**e**) Kaplan–Meier plots of the overall survival of The Cancer Genome Atlas (TCGA) patients with several different cancers grouped according to miR-365a-3p expression. (**f**) Box–whisker plot of the results of the growth of xenograft human tumor cells (SKOV3.ip1) grafted into mice and treated with anti-miR-365a-3p incorporated into dioleoylphosphatidylcholine (DOPC) nanoliposomes.

Multiple pathways in our screen were affected by expression miR-365a (Figure 5). MiR-365a expression is upregulated in breast tumors and is a potential circulating marker for estrogen receptor-positive breast tumors.^15^ Previous studies demonstrated that miR-365a targets BCL2 and cyclin D,^16,17^ NKX2-1,^18,19^ and HDAC4.^20^ In the present study, we found that miR-365a functional regulated steroid hormone, EMT, cell cycle, PI3K/AKT, and MAPK pathways. Given the observed pleiotropic effect of miR-365a, we performed gene ontology-based analysis of targets of miR-365a that are bound by argonaute in argonaute cross-linked immunoprecipitation and next-generation sequencing (AGO-CLIP-seq) cell line experiments^21^ and found a strong association with chromatin modification pathways (Figure 6a,b). Experimental analysis of changes in mRNA expression demonstrated that overexpression of miR-365a in MDA-MB-231 and SKOV3.ip1 cells resulted in downregulation of expression of mRNAs for many chromatin modifiers (Figure 6c) and coordinated upregulation of histone H3 acetylation (Figure 6d).

We performed a survival analysis to determine whether miR-365a expression is associated with cancer patient clinical outcomes. Expression of miR-365a in several cancer types in The Cancer Genome Atlas data was markedly associated with poor patient survival (Figure 6e). Previous studies and our present results demonstrated miR-365a involvement in numerous critical functional pathways underscored the importance of this miRNA in cancer. We thus examined the functional consequences of miR-365a expression on the growth of tumor cells *in vivo*. Treatment with miR-365a antimir (anti-miR-365a) incorporated in dioleoylphosphatidylcholine nanoliposomes of SKOV3.ip1 tumor-bearing mice resulted in marked reductions in tumor weight and nodule number (Figure 6f), which is consistent with miR-365a having a major role in tumor progression.

## Discussion

miRNA regulation of gene expression is important in determining cell fate and maintaining cellular homeostasis.^22^ In the present study, we grouped 879 miRNAs into five clusters based on their similarity in regulating phosphoprotein and total protein levels. The miRNAs in cluster 2 downregulated cell cycle protein levels, inhibited cell proliferation, and had the most consistent functional effects across the three cell lines. Many cluster 2 miRNAs, such as miR-124 and miR-34, are implicated as tumor suppressors by inhibiting cell cycle progression and proliferation.^7,23^ By focusing on the functional regulation of critical proteins in important pathways, we identified miRNAs with similar functional effects and shared tumor-suppressive qualities.

Analyzing changes in the phosphorylation levels for signaling proteins induced by miRNA perturbations, we were able to infer connectivity among constituents of cellular signaling networks (Figure 3). The inferred networks captured many well-known molecular links as well as novel, cell line-specific and mutation-specific network interplay. Furthermore, we identified miRNAs responsible for modulating activity in these inferred pathways (Figure 4).

A recent study demonstrated that miRNAs regulate mRNAs predominantly via transcript destabilization and degradation.^24^ However, mRNA-level studies cannot capture the indirect effects of miRNAs on protein levels owing to translational inhibition as well as indirect or integrative effects on protein stability or post-translational modification. Indeed, from our RPPA screen we observed that each miRNA produces a marked change in the abundance of about 20% of the total proteins measured (Supplementary Figure 7), more than what would be expected by direct targeting. The mechanisms and roles of miRNAs in various diseases will become clearer and more comprehensive as novel methods of proteomic profiling of the effects of miRNAs emerge and become robust.^25–28^

We have shown that miRNAs modulate protein levels, signaling pathways and cell cycle progression. We identified miR-365, miR-555 and miR-665 as important regulators of multiple cellular pathways—miR-365 being previously implicated in cancer pathophysiology.^15,17,18,29^ Our analysis demonstrated that miR-365 exerts pleiotropic effects by targeting multiple gene transcripts including regulatory genes, such as chromatin modifiers. Interestingly, the clustering of miR-365a with other cluster 2 miRNA is likely due to its regulation of cell cycle proteins. However, the proliferation of MDA-MB-231 and SKOV3.ip1 cells is not decreased by miR-365a in 2D cultures (Supplementary Figure 8), unlike the majority other cluster 2 miRNAs. MiR-365a regulates many pathways besides the cell cycle owing to its high level regulation of genes via regulation of chromatin modifiers. The effect of miR-365a upregulation may be context specific as demonstrated by the opposite association with survival in uterine cancer patients and differential association with survival in breast cancer subtypes.

Our unbiased systems level analysis of miRNA-induced changes in the levels of a select set of proteins and phosphoproteins demonstrated that a single robust proteomic screen can both recapitulate much of the previously demonstrated biology and provide important insight into new miRNA-protein interactions and regulatory features of cancer signaling pathways. The data presented herein include those on numerous biologically significant miRNA-induced alterations in different pathways that can be further examined in detail for the identification of potential clinical biomarkers of cancer or miRNAs as therapeutics. With the recent initiation of clinical trials of miRNAs,^30^ understanding the functional effects of miRNAs on biological pathways will be important in successfully capitalizing on the promise of this novel technology.
