# Supplementary material for: Genome-wide perturbations by miRNAs map onto functional cellular pathways, identifying regulators of chromatin modifiers
Source: NPJ Syst Biol Appl. 2015 Sep 28;1:15001–. doi: 10.1038/npjsba.2015.1 (PMC5516802; doi:10.1038/npjsba.2015.1)
Supplement: Supplementary Methods and Legends [file npjsba20151-s1.doc]

**Supplementary Methods**

#### RPPA

The miRNA library screen (synthesized by Dharmacon) was performed on the reverse-phase protein array (RPPA) platform as follows. MDA-MB-231, SKOV3, and HEYA8 cell lines were seeded into the wells of 96-well plates at 5,000 cells per well. The cells were transfected with 50 nM miRNA and after 48 hours lysates were prepared as previously described [^1^](#_ENREF_1). Serially diluted cell lysates were transferred to nitrocellulose-coated FAST slides (Whatman) and each slide was probed with a primary antibody and a biotinylated secondary antibody. Signal was detected using a DAB colorimetric reactions and spot densities were determined using MiroVigene software package (<http://www.vigenetech.com/>). Protein concentrations were defined by fitting the sample dilutions by Super Curve Fitting (“Supercurve Fitting” developed by the Department of Bioinfomatics and Computational Biology in MD Anderson Cancer Center, “http://bioinformatics.mdanderson.org/OOMPA”). The protein concentrations were normalized by median centering across proteins and samples. Screen data is available at http://app1.bioinformatics.mdanderson.org/tcpa/_design/basic/download.html under the accession number TCPA00000001.

#### Cell Proliferation

MDA-MB-231 cells were transfected with miRNA mimics in our miRNA library as above. After 48 hrs of growth the relative proliferation was determined by adding 5ul of Cell Titer Blue Cell Viability Assay Reagent (Promega) to each well and incubate at 37°C for 2 hours. The resulting fluorescence was measured at 530nm Ex / 604nm Em.

### Statistical Analyses

#### Concensus Clustering

MiRNA were clustered by the changes elicited on protein levels in screen1 using only the 103 proteins common to the first and second screen. Consensus clustering was performed using R package ConsensusClusterPlus v1.18.0 [^2^](#_ENREF_2) with maxK = 15, 100 repititions and exlusion of 10% of items and variables during each iteration. Variable importance was measured using random forest classification. Each consensus cluster was classified relative to all other consensus clusters. Variable values were permuted and the out-of-bag (OOB) error rate was determined for each protein after permutation.

### *De novo* network estimation

*De novo* cell-line-specific phospho-protein signaling networks were inferred using statistical models known as Gaussian graphical models [^3^](#_ENREF_3). These models describe probabilistic relationships between variables using an undirected graph or network. Interplay between nodes is considered within a global, multi-dimensional framework through calculation of partial correlations, which quantify association between pairs of nodes while accounting for the effects of all other observed nodes. The number of parameters in these network models is large relative to the sample size (here, number of miRNA), resulting in the potential for over-fitting. Therefore, we estimated the networks using an l_1_-penalized approach known as graphical lasso [^4^](#_ENREF_4) that learns sparse networks. A parameter that controls the strength of the regularization, and therefore the sparsity of the resulting networks, was set (individually for each network) using the Bayesian information criterion (BIC). Since the units for each protein are arbitrary (depending on affinity and avidity of specific antibodies), for each cell line the data for each protein were log transformed and then standardized to have zero mean and unit variance prior to network inference.

### Western Blot Analysis

Cells were treated with miRNA or drugs in 96-well plates. The cells were lysed and proteins separated by PAGE then transferred to nitrocellulose membranes. Membranes were probed with primary antibodies and detected with HRP-conjugated secondary antibodies.

### TCPA protein correlation

RPPA measurements of TCGA pancan11 samples were downloaded from the The Cancer Protein Atlas (TCPA) website (<http://app1.bioinformatics.mdanderson.org/tcpa/_design/basic/index.html>). Pearson’s correlation tests between phospho-MEK and phospho-GSK3 or phospho-ERK and phospho-GSK3 were performed using the R statistical computing environment.

### Network visualization of miRNA-protein interactions

Interactions between miRNA and (phospho-)proteins in the screens were defined by the impact a miRNA had on a protein over control miRNA (see discrete data above). The miRNA-protein interactions were used to create networks using Cytoscape v3.1.0[^5^](#_ENREF_5).

#### Mouse xenograft tumor model

Twenty mice were given intraperitoneal injections of SKOV3 cells (10^6 cells per mouse). After 7 days the mice were treated with intraperitoneal injections of miRNA encapsulated in liposomes (5 μg/ mouse in 200 μl of PBS) twice a week until mice became moribund in either group. Half of the mice were treated with a control miRNA hairpin inhibitor and the other half were treated with the miR-365a hairpin inhibitor. The mice were sacrificed 30 days after the initial injection and the mouse body weight, tumor weight, tumor nodule count, location of metastases, and presence of ascites was measured.

Data were normalized

#### Data normalization

Data were normalized to correct for plate to plate variation(3). For MDA-MB-231 screen 1, the expression levels of proteins were normalized with respect to the mean of the expression levels for the controls (mock and negative). This gives the fold change data FPij for observation i and protein j for j=1,…,n where n is the number of proteins. The discrete data can then be computed as follows:

For antibody j, compute the fold change standard deviation FSTDj for the fold change data of the 44 controls. If | FPi,j –1 | > 2.33×FSTDj, then expression level is 2% significantly low/high.

The data can be converted into discrete form by setting Discrete Protein expression:

DPi,j = -1, if | FPi,j –1 | ≤ -2.33×FSTDj

DPi,j= 0, if | FPi,j –1 | > -2.33×FSTDj and | FPi,j –1 | < 2.33×FSTDj

DPi,j = 1, if | FPi,j –1 | ≥ 2.33×FSTDj

For the cell lines in the second screen, the fold change of protein expression levels can be determined as above, but due to the lower number of controls, the standard deviation of the controls from the first screen was used to transform the continuous fold change data into discrete data as above.

#### Support Vector Machine

SVM analysis was used to predict proliferation from expression levels of proteins. Since the number of proteins is large, we applied principle component analysis (PCA) to reduce the dimension of the data. After reduction of this data dimension, we classified proliferation into 2 classes: Group 1 and Group 2. Group 1 consists of all miRNAs for which proliferation values are greater than the lowest 5% of control proliferation values. The rest, for the initial classification, become part of Group 2. By applying the leave one out method, and testing the SVM model using SVM-light and Radial basis as Kernel, we found a test error of greater than 20% and hence no good SVM model fitted this data well. We therefore sub-divided group 2 to obtain 3 classes: ‘normal-proliferation group’ (group 1), medium-low-proliferation group (group 2) and low-proliferation group (group 3). We tried six-different divisions as below:

1, the samples of lowest 5% proliferation are in the “low” group, others except the “normal” samples in “medium low” group.

2, the samples of lowest 10% proliferation are in the “low” group, others except the “normal” samples in “medium low” group.

3, the samples of lowest 20% proliferation are in the “low” group, others except the “normal” samples in “medium low” group.

4, the samples of lowest 30% proliferation are in the “low” group, others except the “normal” samples in “medium low” group.

5, the samples of lowest 40% proliferation are in the “low” group, others except the “normal” samples in “medium low” group.

6, the samples of lowest 50% proliferation are in the “low” group, others except the “normal” samples in “medium low” group.

We then trained and tested on only group 1 (normal) and group 3 (low). In order to estimate the model feasibility, we computed the confusion matrix as below:

1, Denote N_N_ and N_L_ respectively the size of “normal” group and “low” group.

2, Count the number of “normal” group classified to class “normal” and “low”, denote as N_NN_ and N_NL_; Count the number of “low” group classified to class “normal” and “low”, denote as N_LN_ and N_LL_.

3, Calculate the confusion matrix as below:

CM=$\left[\begin{matrix} N_{NN}/N_{N} & N_{NL}/N_{N} \\ N_{LN}/N_{L} & N_{LL}/N_{L} \end{matrix}\right]$

$N_{NL}/N_{N}$ and $N_{LN}/N_{L}$ gives the relative error for each group.

The results showed that the 2^nd^ division, lowest 10% (low group, size=88) as group 3, gives the smallest test error (Figure 2D). The average test error=7.5%, which is acceptable.

#### Transistion Matrix Analysis

To compare the stability between the screens for the same cell line we used a transition matrix analysis. For the discrete data of screen i, i=1, 2, we computed the numbers NB_i_, NR_i_ and NW_i_ for data points with value -1 (blue), 1 (red) and 0 (white) respectively. The transition matrix then computes the proportions from blue/red/white in screen i to blue/red/white in screen j. Let BB denote the number of blue to blue, BR: blue to red, BW: blue to white, RB: red to blue, RR: red to red, RW: red to white, WB: white to blue, WR: white to red, and WW: white to white. The transition matrix T_ij_ from screen i to screen j is then given as below:

T_ij_ = $\left[\begin{matrix} BB/NB_{i} & BR/NB_{i} & BW/NB_{i} \\ RB/NR_{i} & RR/NR_{i} & RW/NR_{i} \\ WB/NW_{i} & WR/NW_{i} & WW/NW_{i} \end{matrix}\right]$

If the diagonals are all close to 1, the miRNA effect of the 2 screens is stable. The percentage of stability from screen i to screen j can then be computed as the ratio (GG + RR + BB)/(NG_i_ + NR_i_ + NB_i_). We get 78% stability from screen 1 to screen 2 for MDA-MB-231 cell line.

**Supplementary Figure Legends**

Supplementary Figure 1. Diagram of the number of miRNA screened by RPPA and the number of proteins in the functional pathways. Each protein is not unique to any one functional pathway.

Supplementary Figure 2. Well-studied miRNA families cluster together by unsupervised hierarchical clustering. Selected miRNA from screen1 were clustered using Euclidean distance and average linkage. The let-7, miR-17-93, and miR-200 families co-cluster based on their similar effect upon the proteins screened.

Supplementary Figure 3. Consensus clustering K selection. (a) Cumulative distribution function (CDF) plots of consensus scores from the K’s sampled to cluster the miRNA in screen1. (b) Change in area under the CDF curve from K-1 to K. K= 5 was chosen by parsimony as it was the smallest K to result in a small relative change in area under the CDF. Plots are output from the R-package ConsensusClusterPlus.

Supplementary Figure 4. Consistency of miRNA-protein regulation across screens and cell lines. (a) Scatterplot of the fraction of up- and down-regulated proteins for each miRNA determined by Markov transition matrix. (b) Fraction of like-transitions from screen1 to screen2. (c) Box-and-whisker plots of relative protein levels changes by miRNA in functional clusters from screen2 and across 3 cell lines. Shown are the top proteins regulated by cluster 2 miRNA (light blue) and cluster 5 miRNA (dark blue).

Supplementary Figure 5. Correlation in fold-change data across screens 1 and 2. Shown are scatterplot representations of the fold-change in protein for matched miRNA perturbations in MDA-MB-231 cells in screen1 and screen2. Each point in the scatterplot represents the miRNA used to perturb the cell and is colored by the consensus cluster to which the miRNA belongs. The Pearson’s correlation estimate (r) is shown for each plot.

Supplementary Figure 6. Protein correlation with cell proliferation. (a) List of potential activators and repressors from RPPA. (b) Display of correlation between proliferation and protein expression level for two proteins.

Supplementary Figure 7. Waffle plot representing the number of possible miRNA-total protein interactions that can be detected in the first screen. miRNA-protein interactions were separated according to whether or not the interactions were predicted by TargetScan. Predicted targets were available for 679 of the 879 miRNA screened. There are 58,394 binary interactions for 679 miRNA and 86 total proteins. Of the total possible interactions, 2,568 are predicted by TargetScan. Shown are the discretized results between predicted/non-predicated targets and 86 total protein levels.

Supplementary Figure 8. Plot of cell proliferation results from SKOV3.ip1 cells transfected with a non-targeting miRNA control, miRNA-365a or a miRNA-365a hairpin inhibitor.

Supplementary Table 1. Details of the number of miRNAs, proteins and the cell lines used in the two screens performed in this study.

Supplementary Table 2. List of antibodies used in this study and their sources.

Supplementary Table 3. List of miRNAs in library used in this study. miRNAs are labeled by their consensus cluster.

**References**

1 Lu, Y. *et al.* Kinome siRNA-phosphoproteomic screen identifies networks regulating AKT signaling. *Oncogene* **30**, 4567-4577, doi:10.1038/onc.2011.164 (2011).

2 Wilkerson, M. D. & Hayes, D. N. ConsensusClusterPlus: a class discovery tool with confidence assessments and item tracking. *Bioinformatics* **26**, 1572-1573, doi:10.1093/bioinformatics/btq170 (2010).

3 Rue, H. v. & Held, L. *Gaussian Markov random fields : theory and applications*. (Chapman & Hall/CRC, 2005).

4 Friedman, J., Hastie, T. & Tibshirani, R. Sparse inverse covariance estimation with the graphical lasso. *Biostatistics* **9**, 432-441, doi:10.1093/biostatistics/kxm045 (2008).

5 Shannon, P. *et al.* Cytoscape: a software environment for integrated models of biomolecular interaction networks. *Genome research* **13**, 2498-2504, doi:10.1101/gr.1239303 (2003).
