# Supplementary material for: Genome-wide perturbations by miRNAs map onto functional cellular pathways, identifying regulators of chromatin modifiers
Source: NPJ Syst Biol Appl. 2015 Sep 28;1:15001–. doi: 10.1038/npjsba.2015.1 (PMC5516802; doi:10.1038/npjsba.2015.1)

Supplementary Figure 1.

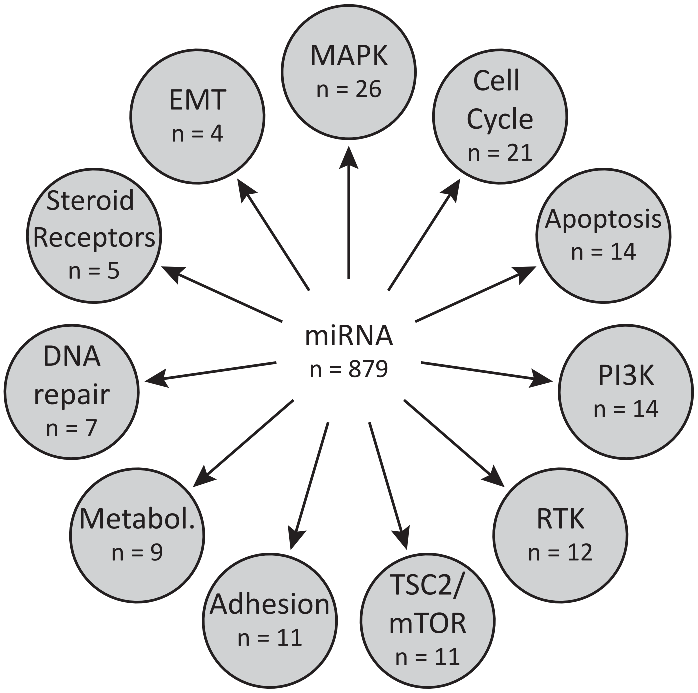

Supplementary Figure 2

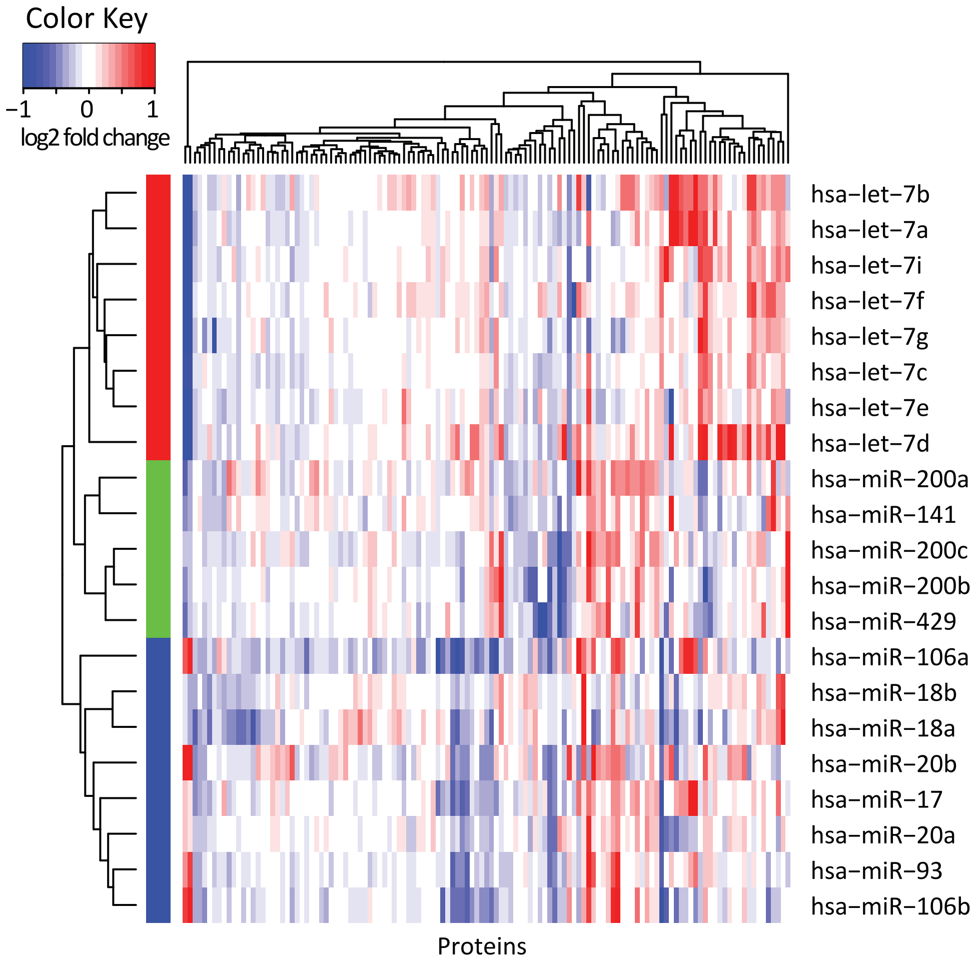

Supplementary Figure 3

(a)

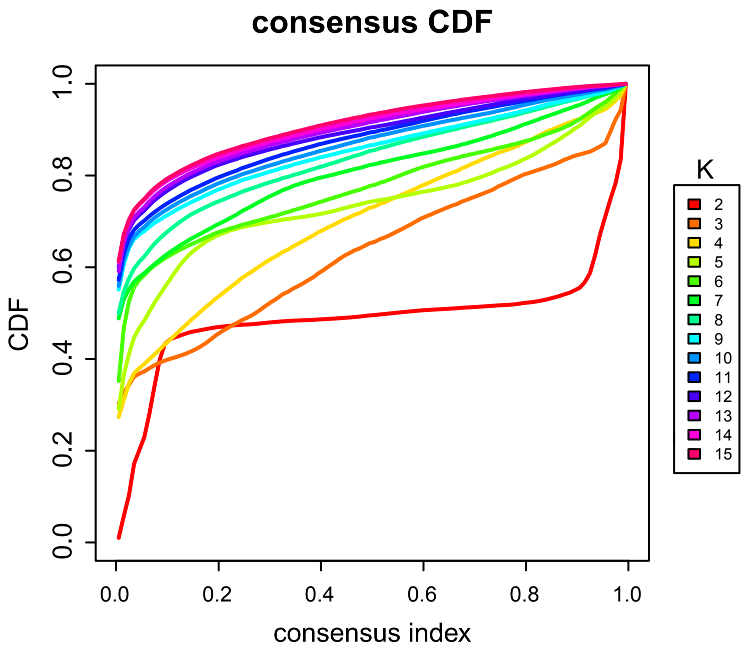

(b)

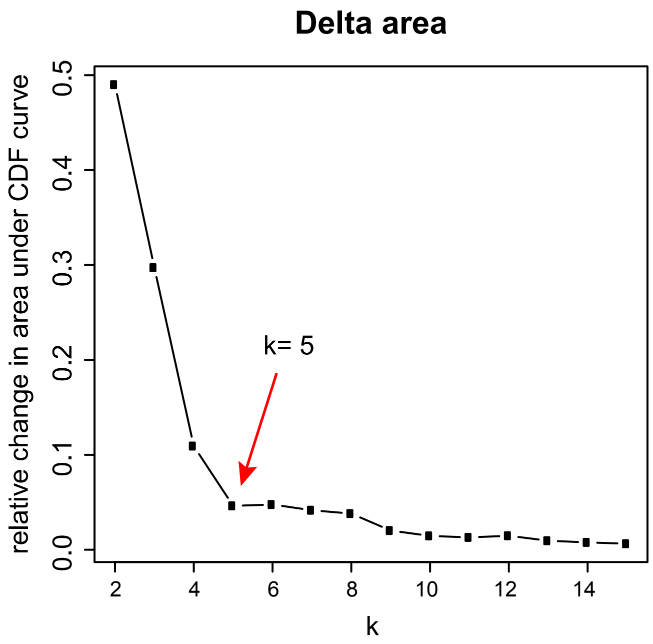

Supplemental Figure 4.

(a)

Average transition matrix from MDA-MB-231 1st to MDA-MB-231 2nd and from MDA-MB-231 2nd to MDA-MB-231 1st.

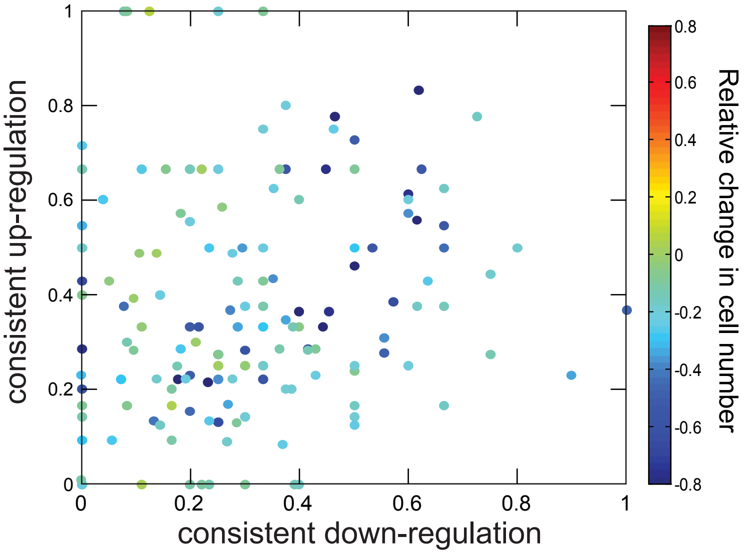

(b)

Transitions across two screens

		Screen2		
		**blue**	**red**	**white**
Screen1	**blue**	0.45	0.02	0.53
	**red**	0.02	0.38	0.60
	**white**	0.07	0.06	0.87

(c)

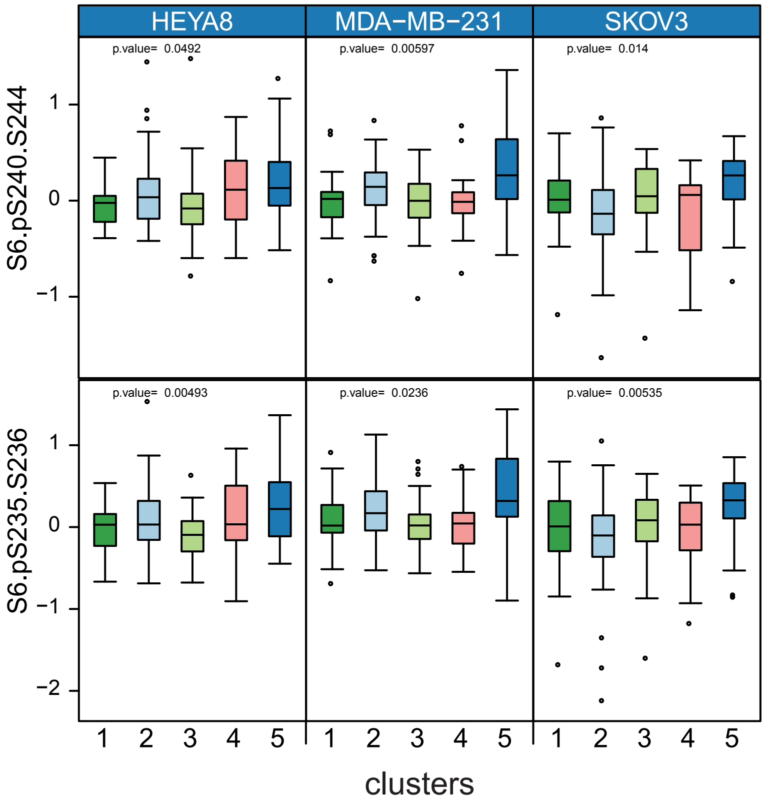

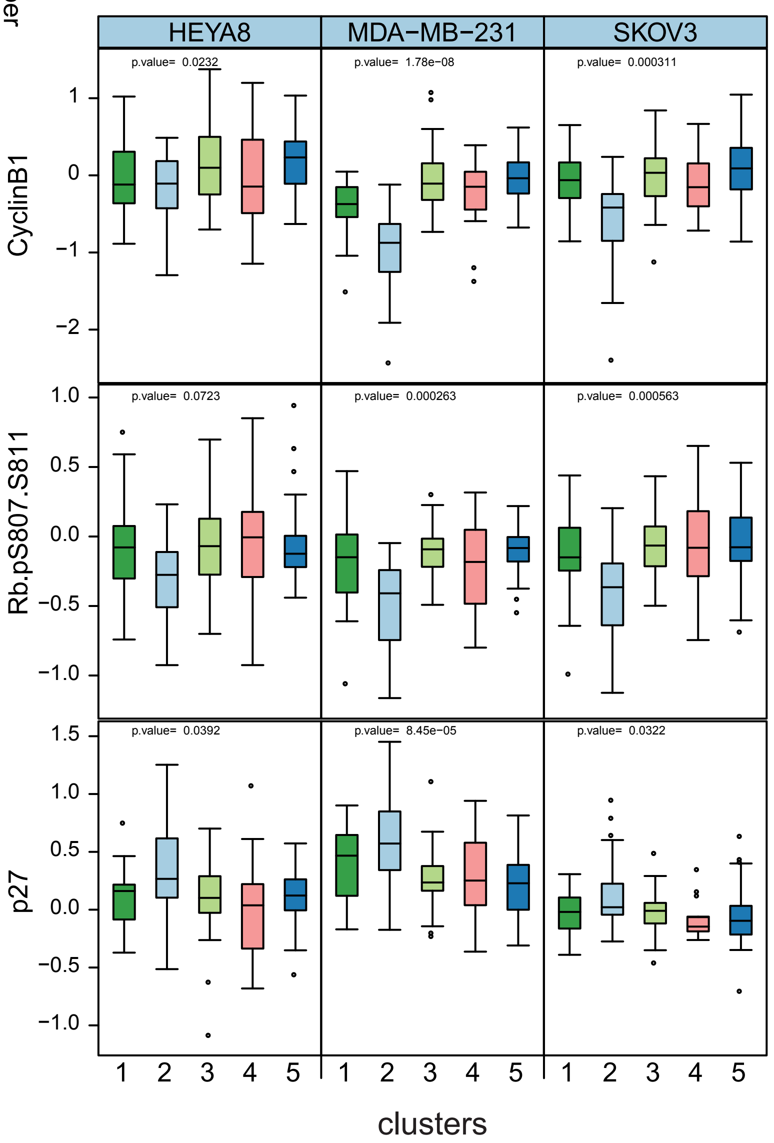

Supplemental Figure 5

Fold-change in proteins across screens 1 & 2.

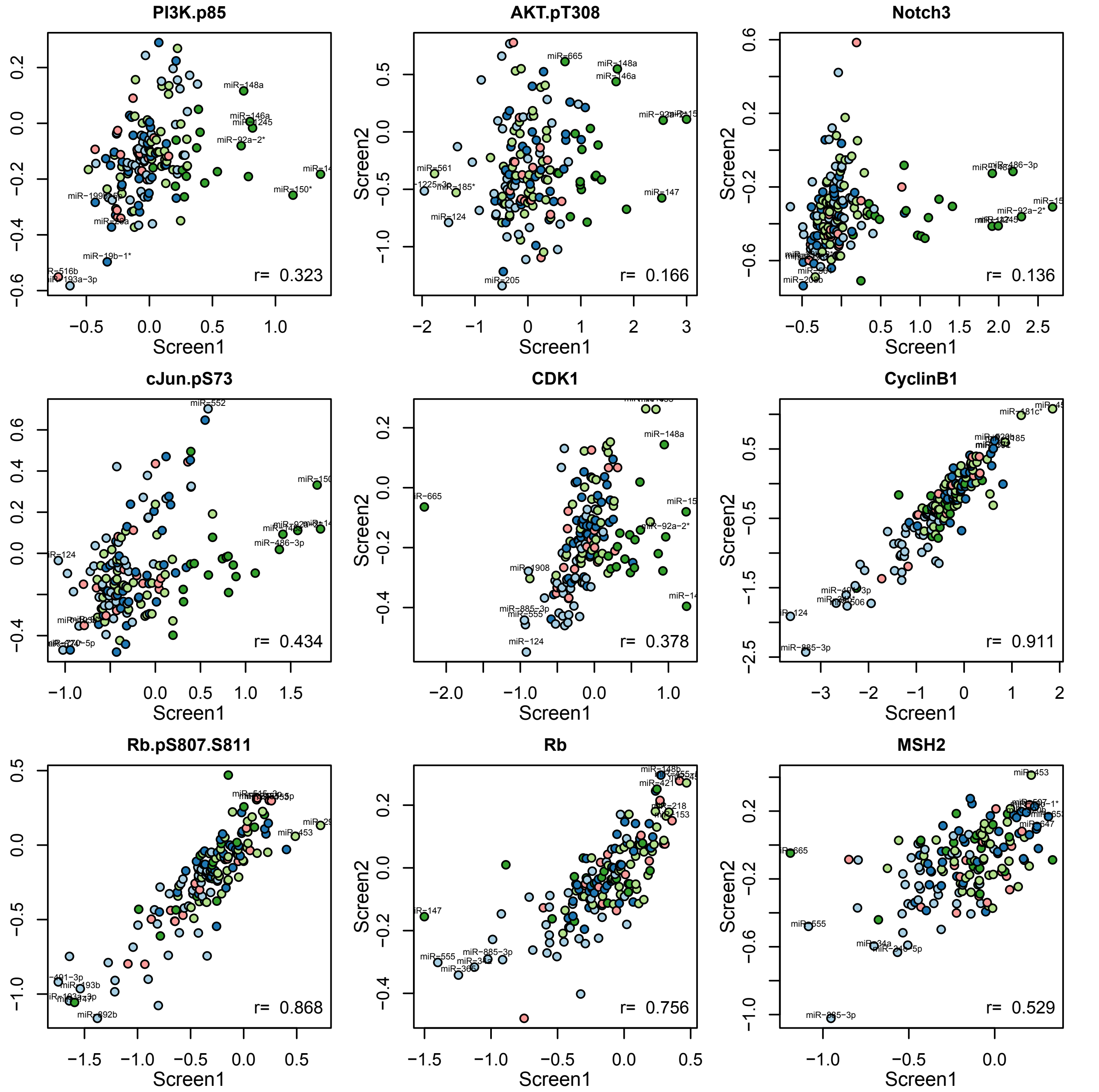

Supplemental Figure 5_continued

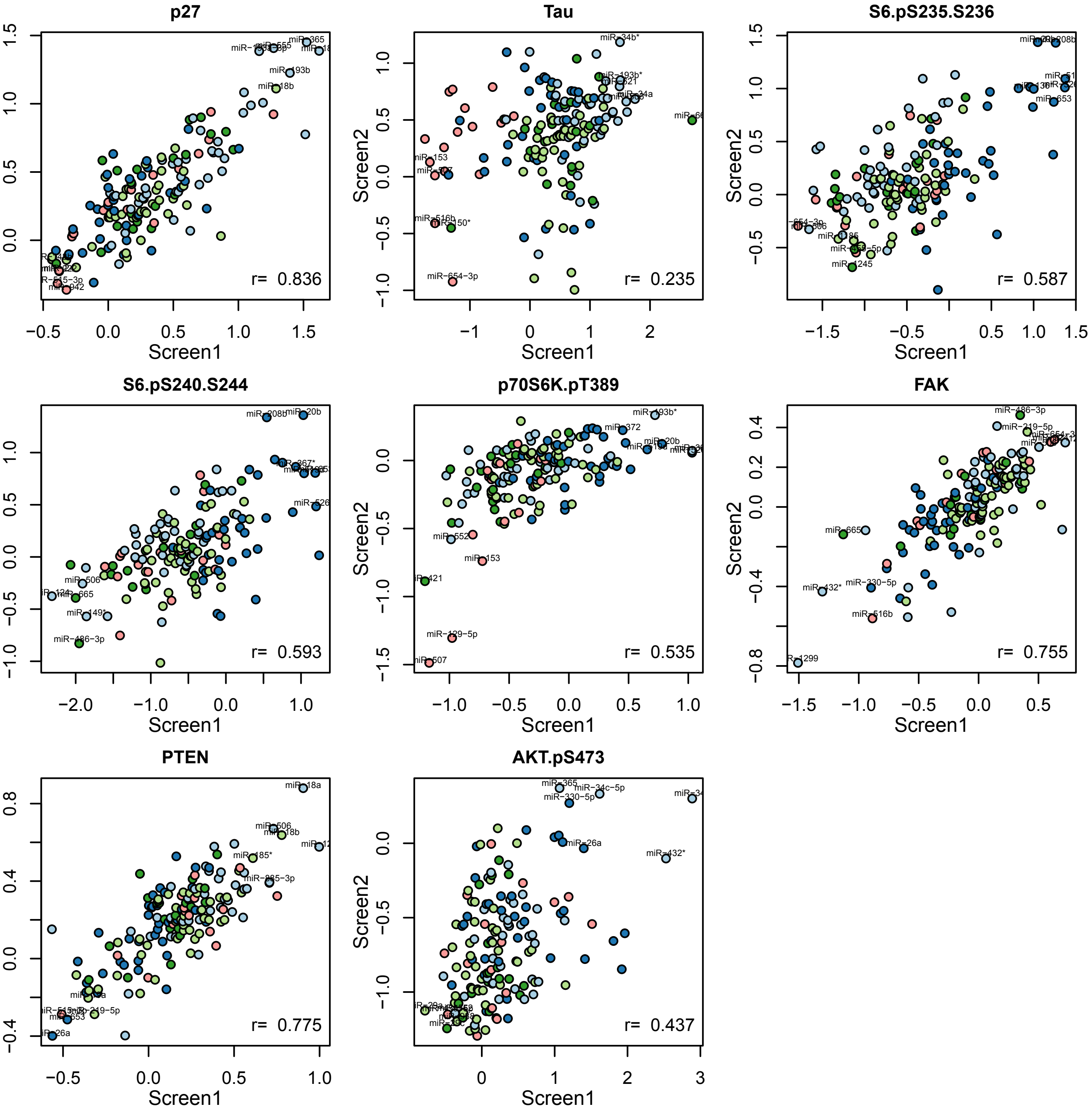

Supplementary Figure 6.

(a)

Potential Activators	Potential Repressors
Rb.pS807.S811	p27
MEK1.pS217	Cyclin E1
c-Myc	p53
Rb	PTEN
4EBP1.pS65	TGM2
MSH2	Tuberin.pT1462
c-Raf.pS388	YAP
Rad51	BIM
Chk1	PR
	Hif1α
	LKB1
	GAPDH
	Tuberin

(b)

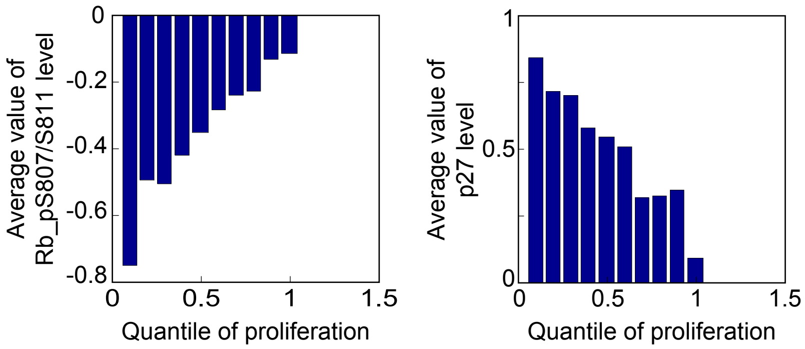

Supplementary Figure 7.

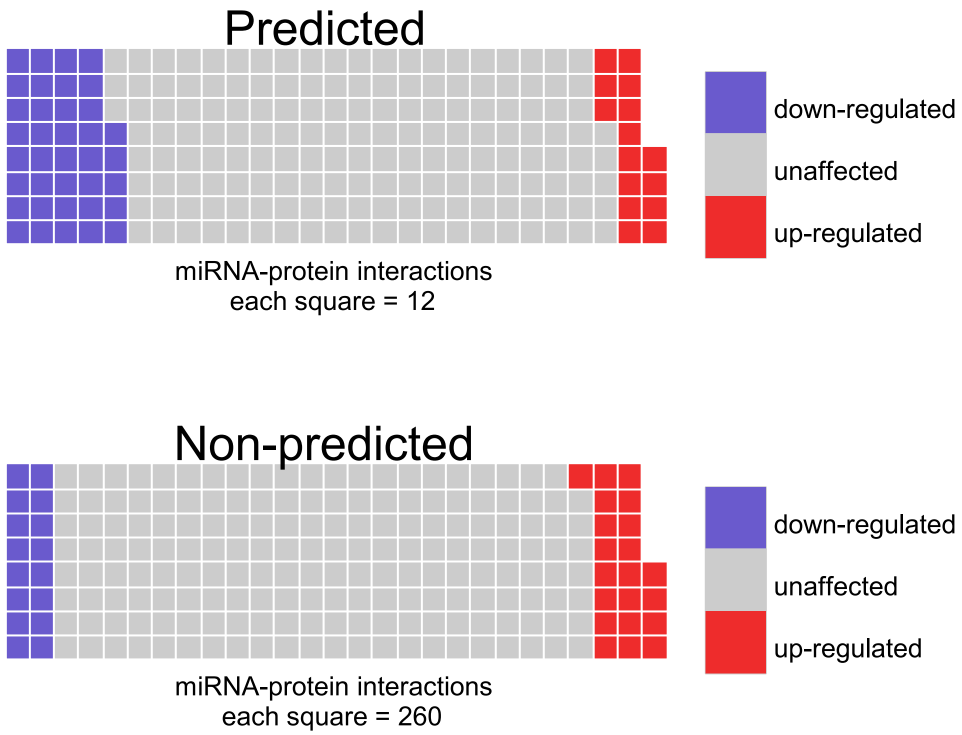

Supplemental Figure 8.

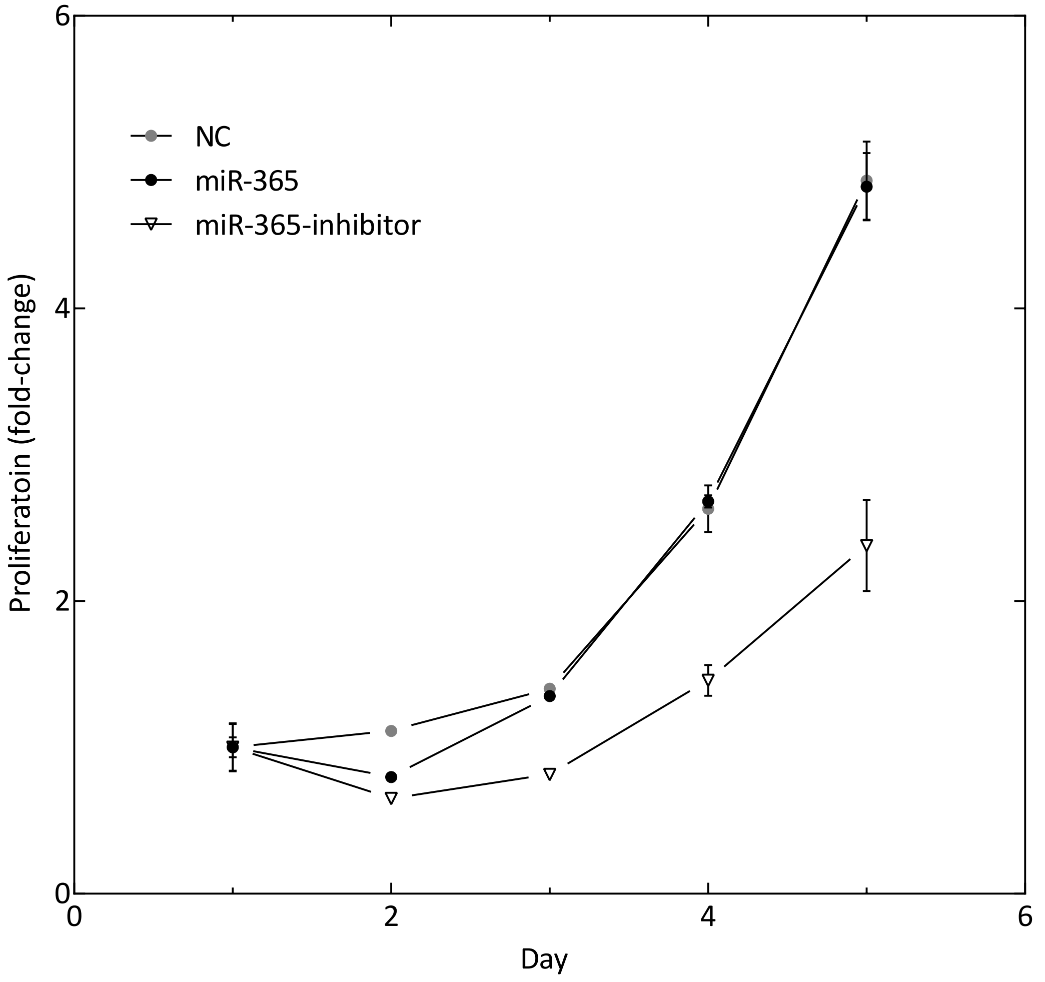

Supplement: Supplementary Figures [file npjsba20151-s2.pdf]
